# Chaperones and Proteostasis: Role in Parkinson’s Disease

**DOI:** 10.3390/diseases8020024

**Published:** 2020-06-22

**Authors:** Neha Joshi, Atchaya Raveendran, Shirisha Nagotu

**Affiliations:** Organelle Biology and Cellular Ageing Lab, Department of Biosciences and Bioengineering, Indian Institute of Technology Guwahati, Guwahati 781039, Assam, India; neha176106109@iitg.ac.in (N.J.); s.atchaya@iitg.ac.in (A.R.)

**Keywords:** neurodegeneration, chaperones, heat shock protein, Parkinson’s disease, synuclein

## Abstract

Proper folding to attain a defined three-dimensional structure is a prerequisite for the functionality of a protein. Improper folding that eventually leads to formation of protein aggregates is a hallmark of several neurodegenerative disorders. Loss of protein homeostasis triggered by cellular stress conditions is a major contributing factor for the formation of these toxic aggregates. A conserved class of proteins called chaperones and co-chaperones is implicated in maintaining the cellular protein homeostasis. Expanding the body of evidence highlights the role of chaperones as central mediators in the formation, de-aggregation and degradation of the aggregates. Altered expression and function of chaperones is associated with many neurodegenerative diseases including Parkinson’s disease. Several studies indicate that chaperones are at the center of the cause and effect cycle of this disease. An overview of the various chaperones that are associated with homeostasis of Parkinson’s disease-related proteins and their role in pathogenicity will be discussed in this review.

## 1. Introduction

Progressive loss of neurons is the most important characteristic of age-associated neurodegenerative diseases [1]. A common feature of these diseases is the accumulation of misfolded or abnormal proteins, some of which have a propensity to aggregate [2]. The incidence of such age-dependent disorders is expected to triple by 2050, and unfortunately, effective strategies that can stop disease progression are currently unavailable. Parkinson’s disease (PD) is a movement-related neurodegenerative disorder and is the second most common after Alzheimer’s disease. It is reported to affect about 1% of the population worldwide [3]. PD usually occurs between the ages of 40–80 and is less common in younger individuals (<5%) [4]. Men are reported to be at higher risk for the disease [5]. The most important area of the brain that undergoes neurodegeneration in this disease is the substantia nigra pars compacta (SNpc) (cells of this area produce the neurotransmitter dopamine) [6]. It is primarily characterized by movement impairment. The early-stage clinical symptoms of the disease are bradykinesia, resting tremor and muscle rigidity (frozen muscle), while in the later stage, patients also experience loss of postural reflex, short shuffling steps and flexed posture. In addition to the above, non-motor symptoms include mood disorder, sensory loss and cognitive impairment [4]. Depending on the cause of the disease, PD is categorized into familial and sporadic types [4]. In 90% of PD cases, no monogenetic cause is reported. An interaction between the genetic and environmental factors leads to a disease in the later stages of life called sporadic PD. Many factors like pesticides, heavy metal toxicity, methamphetamine (METH), tobacco and alcohol are reported to cause sporadic forms of the disease. PD passed on genetically from family members is known as familial PD and accounts for 5–10% of all PD cases [7].

Multiple cellular pathways and mechanisms linked to PD such as protein misfolding and aggregation, proteasome impairment, lysosome-autophagy impairment, mitochondrial dysfunction, oxidative stress, calcium homeostasis, reactive oxygen species (ROS) imbalance, axonal transport, neuroinflammation, dysregulated interorganellar crosstalk between ER–mitochondria and mitochondria–lysosome, etc. are extensively studied [8,9]. α-synuclein (*SNCA*/PARK1), Parkin (*PRKN*/PARK2), PTEN-induced putative kinase 1 (*PINK1*/PARK6), leucine-rich repeat kinase 2 (*LRRK2*/PARK8), DJ-1 (PARK7), vacuolar protein sorting-associated protein 35 (*VPS35*/PARK17), ATP13A2 (PARK9) and glucocerebrosidase (GBA) are some of the extensively studied proteins for their association with PD [4]. The cytopathological marker of PD is the formation of specific cytoplasmic proteinaceous inclusions called Lewy bodies (LBs) in neurons. LBs are predominantly composed of misfolded α-synuclein [9]. 

As mentioned earlier, abnormal protein folding or impairment of the clearance of potentially cytotoxic protein species is a major feature that contributes to PD. This hampered proteostasis can be modulated by molecular chaperones that prevent aggregation of unfolded or misfolded proteins and facilitate their refolding [10,11] and that aid in protein degradation [12,13,14]. Extensive crosstalk between the chaperones is also reported. This crosstalk and the activity of both constitutive and inducible chaperones is the requisite for maintaining protein homeostasis [15]. In this review, we provide an overview of chaperones involved in the proteostasis and regulation of major PD associated proteins (Table 1).

## 2. α-Synuclein: Folding, Higher-Order Structures and Degradation

**Figure 1:**
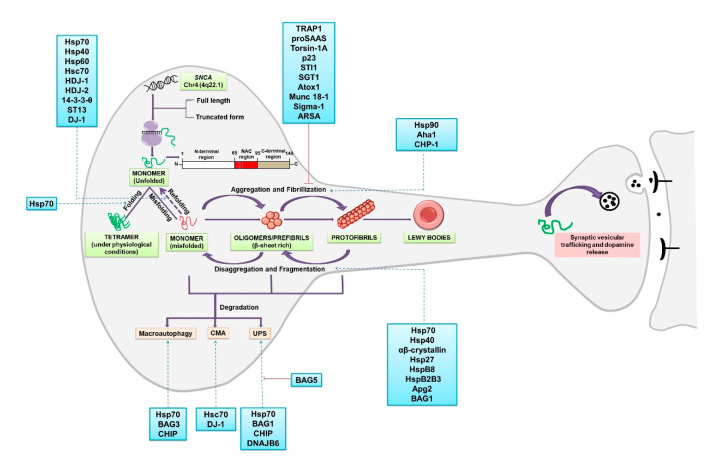
α-synuclein folding, oligomerization and aggregation: SNCA gene on chromosome 4 encodes for the protein α-synuclein. Two truncated forms of the proteins of 126aa and 112aa are also found in the brain together with the full length. The structure of the protein comprises of three distinct domains: amphipathic N-terminus (1–60 residues), a central NAC region (61–95 residues) and the C-terminus region (96–140) [192]. A role for α-synuclein in the membrane curvature and interaction with the SNARE complex and, therefore, in synaptic vesicular trafficking and the release of dopamine have been reported [193]. Two important facets of α-synuclein proteostasis involve chaperone-dependent regulation of protein folding and degradation of the misfolded protein. α-synuclein monomers interact and form a stable homotetrameric structure under physiological conditions [194], whereas in pathological conditions, upon misfolding, the monomer is converted into β-sheet rich oligomers or prefibrills. The oligomers ultimately change into highly ordered fibrils which subsequently form LBs [16]. Chaperone-aided fragmentation of the fibrils and disaggregation have also been reported [39]. Degradation of the misfolded protein can occur by three different mechanisms: proteasome-assisted UPS (Ubiquitin Proteasome System), chaperone-mediated autophagy (CMA; Lamp2A mediated) and macroautophagy, both dependent on lysosome [10]. Several chaperones and co-chaperones are identified that modulate various stages of proteostasis of α-synuclein (listed in the boxes). The red line indicates inhibition.

**Figure 2:**
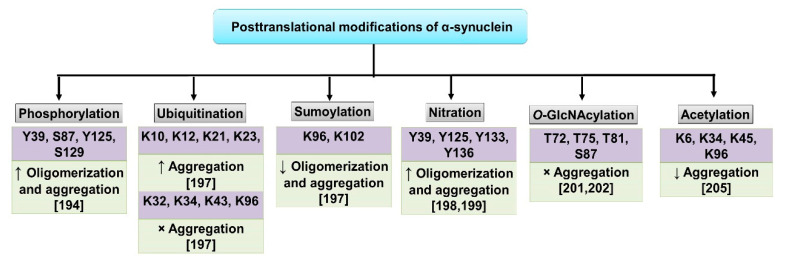
Posttranslational modifications identified that regulate α-synuclein function: Several posttranscriptional modifications (PTMs) have been reported that regulate proteostasis of α-synuclein such as phosphorylation, ubiquitination, sumoylation, nitration, acetylation and *O*-GlcNAcylation. α-synuclein is reported to be phosphorylated at serine (S129 and S87) [195] and tyrosine (Y125, Y133 and Y135) residues [196]. α-synuclein associated with Lewy bodies (LBs) is majorly phosphorylated at S129 [197]. Phosphorylation is catalyzed by several kinases such as Casein kinases (CKs), Polo-like kinases (PLKs) and G-protein coupled receptor kinases (GRKs) [25]. α-synuclein is ubiquitinated by the E3 ubiquitin-protein ligases like C-terminus of Hsp70 Interacting Protein (CHIP), Seven In Absentia Homologue (SIAH) and Nedd4 at various residues that either increase or stop the aggregation of the protein [198]. SUMOylation (SUMO: small ubiquitin-like modifier) of α-synuclein at residues K96 and K102 by PIAS2 that reduces oligomerization and aggregation was reported [199]. Nitration of tyrosine residues Y39, Y125, Y133 and Y136 results in increased oligomerization and aggregation of the protein [200,201], Enhanced fibrillization was also reported upon nitration of Y39 [202]. *O*-GlcNAcylation on the other hand inhibits toxicity caused by extracellular α-synuclein aggregates [203,204]. α-synuclein under physiological conditions is reported to undergo N-terminal acetylation. This modification most likely keeps the protein in its native form and prevents/reduces aggregation. K6, K34, K45 and K96 are reported to undergo acetylation [205]. Several of these PTMs may influence toxicity and aggregation of the protein. α-synuclein may also have several of the abovementioned PTMs at a given time in vivo. ↑—increase, ↓—decrease and ×—stops.

A family of small naturally unfolded proteins extensively studied for their role in neurodegeneration are called synucleins. Among the three members of the family (α-, β- and γ-) α-synuclein is the most extensively studied and is associated with PD [16]. LBs, the pathologic markers of PD, are predominantly composed of α-synuclein, which is a 140-amino-acid-long protein with a molecular mass of 14.4 kDa [17]. α-synuclein is a soluble and unfolded protein that forms tetramers under physiological conditions in the brain (Figure 1 [18,19]). It has a three-domain architecture that comprises the N-terminal region (1–60 residues) that acquires amphiphilic α-helical structure upon lipid binding, a central hydrophobic NAC (Non-Amyloid-β Component) region (61–95 residues) required for filament formation and the carboxy-terminus region reported to exhibit chaperone-like activity [20]. The physiological function of α-synuclein is still not very well understood. Interaction with the SNAP Receptor (SNARE) complex and a role in the release of neurotransmitters has been reported (Figure 1 [16]). Mutations in *SNCA* (duplication, triplication and point mutation) are one of the major contributors to the pathogenesis of PD [21]. Most commonly identified α-synuclein mutations in autosomal dominant PD are A30P, A53T and E46K [22,23,24]. Regulation of the protein via posttranslational modifications (PTM) is also extensively studied. PTMs such as ubiquitination, sumoylation, phosphorylation, nitration and acetylation have been reported with varying effects on oligomerization, aggregation and toxicity (Figure 2; for a detailed review, we recommend reading Reference [25]).

### 2.1. α-Synuclein: Chaperones Involved in Misfolding, Aggregation and Disaggregation

α-synuclein is abundantly expressed in brain cells and comprises 1% of the total cytosolic protein content [16]. Aggregation of misfolded α-synuclein is the first step of the onset of PD pathogenesis. Aggregation starts when the hydrophobic NAC region of α-synuclein undergoes a conformational change into β-sheets, resulting in dimerization and subsequent polymerization forming oligomers and fibrils [26] (Figure 1). Aggregation of the protein is proposed to occur in various phases: the lag phase (very slow growth rate), where monomers and an oligomer both coexist, followed by the exponential (elongation) phase, where a dramatically increased growth rate of β-structured fibrils was observed [25,27]. Several chaperones involved in protein folding, misfolding and aggregation of α-synuclein will be discussed in this section (Figure 1).

In mammals, two forms of Hsp90, the stress-induced Hsp90α and constitutive Hsp90β, are present. Hsp90 is predominantly a cytosolic chaperone, but homologues of the protein in other compartments such as ER (GRP94) and mitochondria (Hsp75 and TRAP1) have also been reported [28]. It is comprised of three domains, N-terminal ATPase domain, middle domain and the C-terminal dimerization domain [21]. It is the predominant chaperone involved in the formation of α-synuclein fibrils in PD [21]. Hsp90 interacts with the NAC region of α-synuclein and promotes fibril maturation in an ATP-dependent manner [29]. Initial studies identified that Hsp90 co-localizes with α-synuclein in the brain of PD patients [30]. Similarly, α-synuclein-induced cellular toxicity was found to be greater in *hsp90 (hsp82)* cells as compared to control cells in the yeast model of PD [31]. Inhibition of Hsp90 leads to decreased oligomerization and fibrillation of α-synuclein in the human H4 neuroglioma cells [32]. Overexpression of TRAP1 (TNF Receptor Associated Protein 1), the homolog of Hsp90 in mitochondria, led to suppressed α-synuclein-induced toxicity in *Drosophila melanogaster*, rat primary neurons and HEK293 cells [33]. Dimerization of Hsp90 leads to conformational changes that are reported to be stabilized by various co-chaperones and ATP [33]. Aha1 (ATPase modulator), a co-chaperone of Hsp90, was reported to stimulate the Hsp90-ATP cycle and to trigger amyloid formation [29]. Interestingly, inhibition of the Hsp90-ATP cycle by p23 and STI1 (Hsp90 co-chaperones) resulted in the formation of soluble α-synuclein oligomers [29,34]. Bohush and colleagues reported an increased level of CHP-1 (co-chaperone of Hsp90) and Hsp90 in LBs of the brain of PD patients, signifying their interaction with α-synuclein [21]. On the other hand, Sgt1, another co-chaperone of Hsp90, was not localized in LBs, suggesting its function as a suppressor of Hsp90 [21]. 

Several chaperones called small Hsps (sHsps) such as Hsp27, Hsp20 and αβ-crystallin were reported to prevent aggregation of α-synuclein by inhibiting the lag and elongation phases of aggregation [35]. Waudby and colleagues reported decreased α-synuclein amyloid concentration due to the binding of αβ-crystallin along the length of α-synuclein fibril [36]. Hsp27 binds to α-synuclein fibrils and decreases its hydrophobicity, which most likely prevents binding of the fibrils to the cell surface [37]. Further, the effect of HspB8 and HspB2B3 chaperones on the fibril formation of wildtype (WT) and mutant variants of α-synuclein was analyzed by Thioflavin T assay and Atomic Force Microscopy (AFM) [38]. Hsp70 interacts with α-synuclein fibrils; however, disaggregation of the fibrils also needs additional factors [39]. Human Hsp70 along with Hsp40 (DNAJ) and Hsp110 were reported to form a disaggregase complex that results in the disassembly or depolarization of α-synuclein fibrils [40]. In vitro solubilization of fibrils by human Hsp70 was increased by the addition of Hsp110 family proteins Apg2/Hsp105α [40]. A similar effect in vitro on α-synuclein fibrils was also reported upon the addition of BAG1 (BCL-2-Associated Athanogene) [40].

Overexpression of yeast Hsp104 results in the disintegration of preformed α-synuclein fibrils in the rat model of PD [41]. TorsinA (homolog of yeast Hsp104) was also reported to colocalize with α-synuclein in LBs of the brain of PD patients. Overexpression of TorsinA, HDJ-1, HDJ-2 and Hsp70 resulted in the inhibition of α-synuclein aggregation in brain cells of PD patients [42]. Interestingly, another neuronal chaperone, proSAAS, was also reported to inhibit the formation of α-synuclein fibrils in humans [43]. The authors reported that proSAAS bound to secreted forms of α-synuclein or intracellular α-synuclein oligomers, reduced further growth and prevented seeding of additional oligomers [43]. Munc18-1 is a protein involved in SNARE complex-mediated neurotransmitter release [44]. Overexpression of Munc18-1 was reported to reduce aggregates of either WT or mutant (A30P) α-synuclein in MKO-PC12 cells [45]. The mutant variant of Munc18-1(EIEE mutants) co-aggregated with α-synuclein in LB-like structures. This was most likely due to the inhibition of the chaperone activity of the mutant protein [45]. Sigma-1 is a non-G protein-coupled receptor that colocalizes to the mitochondria-associated endoplasmic reticulum (ER) membrane (MAM). A reduced level of this protein was reported in the brain of PD patients [46]. Knockdown of the Sigma-1 receptor in mice resulted in enhanced phosphorylation and aggregation of α-synuclein, leading to the death of dopamine-producing neurons in mice [47].

Altered metal ion homeostasis, importantly that of copper (Cu) ion, was reported as a significant factor that contributes to the pathogenesis of PD [48]. Cu ions were reported to have the largest effect in vitro in the acceleration of α-synuclein amyloid formation when compared to other metal ions. Cu ion was reported to interact with the N-terminal of α-synuclein and to promote amyloid formation [49]. Overexpression of the cytoplasmic Cu chaperone Atox1 resulted in the prevention of α-synuclein amyloid formation in human neuronal cell lines [50]. Atox1 is a single domain protein that has Cu-binding sites. In vitro studies revealed that interaction of Atox1 with α-synuclein blocked amyloid formation [51]. A substantial decrease in total Cu in the substantia nigra has been reported in PD patients when compared to healthy adults [52]. The authors suggest a twofold effect of this decrease: reduced activity of the Cu-dependent antioxidant SOD and reduced chaperoning by Atox1, both conditions that can promote PD [50]. 

Arylsulfatase A (ARSA) is a lysosomal hydrolase and is reported to interact with α-synuclein and regulates its proteostasis. Deletion of ARSA led to increased aggregation of α-synuclein in PD models. Pathogenic (L300S) and protective (N352S) mutations in ARSA that vary in their binding affinity to α-synuclein and linked to PD are reported in this study [53]. 

### 2.2. α-Synuclein: Chaperones Involved in Refolding

Molecular chaperones regulate proteostasis either by folding the newly synthesized proteins or by aiding refolding of the nonfunctional, misfolded proteins [10]. Hsp70 reduces α-synuclein aggregation either by aiding refolding or by degradation of the misfolded protein via the UPS (Ubiquitin Proteasome System) and ALP (Autopaghy Lysosomal Pathway). This is a large family of chaperones and comprises several proteins such as HspA1A, HspA1B, HspA6, HspA8 (Hsc70), HSPA9 (the mitochondrial mtHsp70/mortalin) and HspA5 (GRP78/Bip) of the ER [54]. Hsp70 is comprised of two main domains: N-terminal ATPase domain and the C-terminal ligand binding domain. Enhanced chaperone activity of the ADP-bound form of Hsp70 than the ATP-bound form was reported [10]. This interaction of Hsp70 with ATP/ADP and substrate proteins is regulated by various co-chaperones. Hsp40, a member of the J domain-containing family of proteins interacts with the N-terminus of Hsp70 and functions as its co-chaperone. This interaction leads to enhanced ATP hydrolysis and formation of the more active ADP-bound form of Hsp70 [10]. Another interesting feature of the Hsp40 family is its specificity towards substrate selection [55]. In vitro studies reported that Hsp40 binds to the misfolded polypeptide and directs it to Hsp70 for further unfolding and refolding [56]. ST13 (Hip) (a co-chaperone of Hsp70) was reported to interact with the N-terminal domain of Hsp70. This interaction led to an enhanced chaperone function of Hsp70 via the stabilization of its ADP-bound form [57]. Negative regulation of the activity of Hsp70 by another co-chaperone BAG1 was reported. This is achieved either by the dissociation of ADP, thereby resulting in the destabilization of Hsp70, or by competing with the co-chaperone Hip to bind to the ATPase domain of Hsp70 [58]. A reduced level of ST13 was reported in the serum of patients suffering from PD [59]. Mutation in the ATPase domain of Hsp70 (K71S) resulted in increased α-synuclein toxicity in a mouse model of PD, indicating that Hsp70 refolding activity was necessary for its protective function [60]. Degeneration of dopaminergic neurons was triggered when human WT α-synuclein was overexpressed in flies. This effect was however reversed by the co-expression of human Hsp70 and α-synuclein and emphasizes the role of Hsp70 in minimizing the toxic effect of α-synuclein [61]. The authors also reported that inhibition of Hsp70 accelerated neurodegeneration in flies with or without human 𝛼-synuclein transgene [61]. As mentioned above Hsc70, a member of the family, has 85% structural similarity to Hsp70. The function of Hsc70 is also dependent and cycles between the ATP- and ADP-bound forms like Hsp70. Hsc70 effectively binds to α-synuclein fibrils and sequesters it in an assembly-incompetent state. Co-chaperones HDJ-1 and HDJ-2 are reported to be required for this process [62].

Hsp60 is a mitochondrial and cytosolic chaperone involved in protein folding and refolding. Hsp60 refolds α-synuclein in an ATP-independent manner and suppresses α-synuclein amyloid formation in vitro [63]. Further studies reported that the orientation of the apical domain (AD) of Hsp60 plays an important role in its function [64]. Yamamoto and colleagues also reported that isolated AD of human Hsp60 (Hsp60 AD) acted as a mini chaperone and could inhibit amyloid formation in vitro [64]. Another important chaperone involved in the refolding of α-synuclein is 14-3-3-θ. It is abundantly expressed in the brain that plays a potential role in folding, refolding and trafficking of several proteins [65]. Overexpression of 14-3-3-θ in a mice model of PD restored the cellular balance of intracellular and extracellular α-synuclein. This is achieved directly via refolding of intracellular α-synuclein or indirectly by releasing aggregated 𝛼-synuclein out of the cell [66]. DJ-1 inhibits the early stage oligomerization of α-synuclein monomers but not the fibril formation as reported in mouse embryonic stem cells [67]. Studies report that DJ-1 interacts with α-synuclein monomers and oligomers both in vitro and in vivo in HEK293 cells. However, PD-associated mutant variants of the protein were reported to lack this interaction [68]. Enhanced expression of DJ-1 also resulted in reduced dimerization of α-synuclein in the yeast model of PD [68].

### 2.3. α-Synuclein: Chaperones Involved in Degradation

Protein degradation is an important process in maintaining the intracellular homeostasis of proteins. Two important cellular pathways required for protein degradation are the ALP and UPS. Autophagy can be further categorized into macroautophagy, microautophagy and chaperone-mediated autophagy (CMA). Degradation of α-synuclein by UPS and via CMA and macroautophagy have been studied extensively (Figure 1) [69,70]. Understanding protein degradation mechanism is of utmost importance especially in cells such as neurons that are postmitotic and accumulate damage caused by intracellular components.

#### 2.3.1. Degradation via Ubiquitin Proteasome System (UPS)

Selective degradation of ubiquitin-conjugated substrates via the 26S proteasome is called UPS [71]. PD-related proteins, such as Parkin and UCH-L1, along with the components of UPS have been implicated in the degradation of misfolded α-synuclein [72]. A prominent role for UPS in degrading cytosolic smaller *α*-synuclein assemblies has been proposed and is most likely the case in young and healthy organisms [72]. As mentioned in an earlier section, Hsp70 can either refold a protein and prevent its aggregation or target the protein for degradation [60]. Hsp70 enhances binding of parkin to α-synuclein, thereby increasing ubiquitination of α-synuclein in vitro [73]. This suggests that Hsp70 can promote the E3 ligase activity of Parkin to degrade aberrant α-synuclein [73]. Another chaperone with an E3 ubiquitin ligase activity that aids the function of Hsp70 is C-terminus of Hsp70 Interacting Protein (CHIP) [74]. CHIP, a co-chaperone of Hsp70, positively regulates degradation of α-synuclein by UPS and negatively regulates the refolding activity of Hsp70 [75]. CHIP-mediated monoubiquitination or polyubiquitination of α-synuclein has been reported [76]. Interestingly, a role for CHIP in the lysosomal degradation pathway has also been reported [77]. The N-terminal tandem tetratricopeptide repeat (TPR) domain, a highly charged central domain and a C-terminal U-box domain are identified in CHIP [74]. The TPR domain of the protein interacts with Hsp70 and Hsp90, and the U-box domain is required for the E3 ubiquitin ligase function [78]. The TPR domain of CHIP is important for proteasomal degradation of α-synuclein, whereas the UPR domain is involved in the lysosomal degradation pathway [77]. In LBs in human brain, CHIP was observed to colocalize with α-synuclein and Hsp70 [77]. Overexpression of CHIP resulted in both reduced levels and inhibition of inclusion formation of α-synuclein in human H4 neuroglioma cells [77]. CHIP-mediated selective targeting of toxic α-synuclein oligomers for degradation was demonstrated using bimolecular fluorescence complementation assay [79]. BAG1, another co-chaperone of Hsp70, aids in the release of the substrate from Hsp70 in mammalian cells by enhancing the ATPase activity of the chaperone [80]. BAG1 interacts with CHIP at its N-terminus (ubiquitin-like domain), promotes ubiquitination of Hsp70-bound α-synuclein and aids in the recruitment of the Hsp70 complex to the proteasome for degradation of α-synuclein [81,82]. However, overexpression of BAG5 antagonizes CHIP-mediated α-synuclein ubiquitination, resulting in the inability of CHIP to suppress oligomer formation [76]. 

Several DNAJ proteins are linked to PD, and a role for DNAJB6, a member of this family in the suppression of α-synuclein aggregation and proteasomal degradation, has been reported [83]. Knockout of DNAJB6 in HEK239 cells resulted in increased aggregation of α-synuclein [83]. Interestingly, this induced aggregation was abolished upon treatment of the cells with a proteasomal inhibitor (MG132) [83]. 

Most of the ubiquitin-ligases are reported for polyubiquitinate α-synuclein; however, there are a few involved in the monoubiquitination of α-synuclein [84]. Seven In Absentia Homologue (SIAH) is one such E3 ubiquitin-ligase reported for monoubiquitinate α-synuclein both in vitro and in vivo [85]. Monoubiquitnation of α-synuclein resulted in aggregate formation in dopaminergic neurons [85]. Enhanced aggregation of the monoubiquitinated mutant variant A53T of α-synuclein when compared to the WT protein was reported [85]. However, the mechanism as to how this monoubiquitination leads to an increase in aggregation of α-synuclein is not known. Later, contrary to this, monoubiquitination-dependent α-synuclein clearance through the proteasome was also reported [86]. In vitro and in vivo interaction of the deubiquitinase USP9X with α-synuclein was observed, and the de-ubiquitination targeted α-synuclein to the lysosome for further degradation [86]. The knockdown of USP9X in the SH-SY5Y cell line resulted in the accumulation of monoubiquitinated α-synuclein and led to aggregation of α-synuclein into toxic inclusions [86]. Interestingly, the degradation of non-ubiquitinated α-synuclein by proteasome was also reported [87]. α-synuclein phosphorylated at Ser-129 was targeted and degraded in the proteasome albeit in a ubiquitin independent way [87].

Studies also revealed that increased levels of α-synuclein could inhibit proteasomal activity [88]. Overexpression of WT and mutant α-synuclein (A53T) in vitro resulted in a change in the composition of the proteasome and inhibition of its activity [89]. Soluble oligomeric and the aggregated form of WT and mutant α-synuclein inhibited the catalytic activity of the 26S and 20S proteasomes in transgenic mice [90]. Chu and colleagues also reported that overexpression of mutant human α-synuclein (A30P) in the rat substantia nigra resulted in a decline in the proteasomal marker (20S proteasome) [91]. Recently, Zondler and colleagues reported that the impairment of α-synuclein degradation by the proteasome was dependent on the cell type investigated [92]. Proteasomal activity was significantly impaired upon overexpression of WT and mutant α-synuclein in dopaminergic cells lines, such as SH-SY5Y and PC12. In contrast, overexpression of WT and mutant α-synuclein in U2OS ps 2042 (Ubi(G76V)-GFP) cells (osteosarcoma cell line) did not affect the proteasome activity [92]. Although the exact mechanism by which mutant α-synuclein inhibits the proteasome activity is still not clear, few studies report that mutant variants of α-synuclein may directly bind to subunits of the 19S or 20S proteasomes and inhibit its activity [93].

#### 2.3.2. Degradation via Chaperone Mediated Autophagy (CMA)

Cytosolic proteins with the specific amino acid sequence KFERQ are unfolded and transported into the lysosome for degradation via CMA. Hsp70, Hsc70 and other cooperating chaperones are required for protein degradation via CMA. CMA recognition motifs have been identified in α-synuclein, and further studies confirmed this to be an important mode of degradation of the protein [20,94]. Accumulation of α-synuclein due to dysfunctional CMA in human neuroblastoma cells was reported [95]. Targeting of α-synuclein to the lysosome following its interaction with Hsc70 requires the type-2a lysosomal membrane protein, Lamp2A. Subsequently, the lysosome-associated Hsc70 (lHsc70) binds to α-synuclein and directs it for degradation [20]. Cuervo and colleagues isolated lysosomes from rat liver and demonstrated that WT α-synuclein was degraded by CMA while PD-associated mutants of α-synuclein (A30P and A53T) inhibited this pathway. The authors proposed that the mutant variants of α-synuclein bind to Lamp2A with higher affinity, making it nonfunctional [69]. Overexpression of WT α-synuclein leads to an increased level of Lamp2A and Hsc70 in the mice model of PD [96]. The susceptibility of α-synuclein to CMA degradation was also reported to be influenced by PTMs such as phosphorylation and acetylation [20]. Noncovalent interaction of α-synuclein with oxidized dopamine results in conformational changes [97]. This dopamine-modified α-synuclein (DA–α-syn) was reported to form toxic aggregates in the neurons that blocked CMA [97]. Vicente and colleagues reported that various forms (monomeric, dimeric and oligomeric) of α-synuclein can bind to the lysosomal membrane. However, only monomers and dimers were imported into the lysosome, suggesting CMA as a mode of degradation to these forms [97]. Interestingly, overexpression of α-synuclein and inhibition of CMA in neuronal cell types also led to increased accumulation of soluble high molecular weight forms of the protein [98,99]. Similarly, increased α-synuclein levels were also reported upon knockdown of Lamp2A or Hsp70 [100,101]. Downregulation of Lamp2A resulted in the accumulation of ubiquitinated α-synuclein inclusions in the substantia nigra followed by the loss of dopaminergic neurons in rat [102]. Several microRNAs (miRNAs) have been identified that target the CMA pathway and therefore affect degradation of α-synuclein. These miRNAs downregulate levels of Lamp2A or Hsc70, thereby reducing the efficiency of CMA [101]. Interestingly, an upregulation of these miRNAs in the brain of PD patients has also been reported [100]. Neuronal cells were also reported to be protected from the toxicity caused by METH by upregulation of Hsc70 expression. Overexpression of Hsc70 decreased α-synuclein aggregation and apoptosis in these cells [103]. 

A recent study highlights the interplay between PD-associated proteins DJ-1 and α-synuclein [104]. Inhibition of α-synuclein aggregation by DJ-1 via regulation of CMA has been reported by the authors [104]. Deficiency of DJ-1 in α-synuclein-overexpressing cells suppressed upregulation of Lamp2A and Hsc70 in SH-SY5Y cells. Degradation of Lamp2A and increased aggregation of α-synuclein was also observed in cells deficient of DJ-1 [104].

#### 2.3.3. Degradation via Macroautophagy

Macroautophagy, commonly referred to as “autophagy”, is responsible for the degradation of larger protein aggregates. It is a nonselective process where the formation of de novo double membrane-vesicles (autophagosome) sequester intracellular components and traffic them to the lysosomes [105,106]. Atg9 was reported to be involved in the membrane formation of autophagosomes, both in yeast and humans [107,108]. The formation of autophagosomes requires two steps of ubiquitination. Firstly, upon conjugation of Atg12 with Atg5, Atg16-mediated targeting of the complex to the autophagosome is observed [109]. The second step requires Atg8 (LC3), which when cleaved at its C-terminus by Atg4 forms LC3-I and is then conjugated to the lipid phosphatidylethanolamine (PE) by Atg7 and Atg3 to generate LC3-II [110]. α-synuclein overexpression (A53T and WT) was reported to significantly increase the levels of the Atg5 complex in SH-SY5Y cells [111]. Interestingly, increased Atg8/LC3 levels in cortical neurons at the later stage of PD was also reported [111]. Additional proteins such as Histone Deacetylase 6 (HDAC6) and BAG3 are also required for the autophagic degradation of protein aggregates in a process called aggrephagy. In HDAC6-mediated aggrephagy, lysine 63-polyubiquitination by Parkin is a prerequisite for the recognition of substrates and translocation to the aggresome (stress-induced protein aggregates) [112]. A role for two additional proteins, p62 and NBR1, that interact with PE-LC3 and with K63-polyubiquitinated chains was identified in HDAC6-mediated degradation of aggregates [113,114]. Colocalization of HDAC6 with α-synuclein and ubiquitin in the brain sections of PD patients was observed using immunohistochemical studies suggesting HDAC6 as a component of LBs [115]. Inhibition of HDAC6 increased the neurodegeneration of dopaminergic neurons and upregulated the levels of α-synuclein oligomers. HDAC6 overexpression in vitro reversed these effects [116]. Dissociation of the Hsp90–HSF1 (transcription factor) complex and the activation of HSF1 depend on HDAC6 and result in the expression of chaperones such as Hsp70 to prevent α-synuclein aggregation [116]. BAG3-mediated degradation was also reported to be dependent on p62 and NBR1. Recognition of the substrate in this pathway by Hsp70 requires both BAG3 and CHIP [117]. The role of ubiquitination in this pathway is not clearly understood and needs further investigation [117]. Colocalization of BAG3 with Hsp70 and LC3 was observed in the neurons of the midbrains of SNCA^A53T^ transgenic mice. Overexpression of BAG3 in these cells resulted in enhanced degradation of α-synuclein via macroautophagy [118]. 

Inside the lysosome, the aspartyl protease cathepsin (CTSD) was reported as an important protease that degrades α-synuclein. Inactivation of CTSD in cells resulted in an increased level of α-synuclein [119]. Accumulation of higher molecular weight α-synuclein oligomers were reported in CTSD knockout mice [120]. Another important lysosomal protein implicated in α-synuclein accumulation and aggregation is the glucocerebrosidase (GBA). GBA mutations are known to cause lysosome dysfunction, and as α-synuclein is also partly degraded via the lysosome, dysfunction of lysosomes results in the loss of proteostasis. Both the loss-of-function and gain-of-function mutations in GBA are reported to be involved in the development of PD [121].

Apart from the above prominent chaperone-assisted degradation mechanisms, a role for proteases such as calpains and neurosin in the cleavage of normal or aggregated forms of intracellular α-synuclein has been proposed [25]. 

### 2.4. Extracellular α-Synuclein

α-synuclein is observed to be both intracellular and extracellular. Interestingly, larger aggregates are also reported to be transported to the extracellular space [122]. Intracellular forms of α-synuclein have been the center of research majorly. However, recent studies also highlight the pathological role of extracellular synucleins [123]. The release of α-synuclein was reported to be independent of cell death. Upon overexpression, α-synuclein was present in the media of cultured peripheral neurons, brain interstitial fluid and cerebrospinal fluid [124]. The mechanism which regulates the secretion of α-synuclein is not clearly understood as typical secretion signals are absent on the N-terminus of the protein [125]. However, extracellular stress and intracellular mechanisms such as neuronal activity are proposed to be involved in this pathway. A role for Hsp70 and DNAJ chaperones in the transsynaptic release of oligomeric α-synuclein has been reported [124,126]. Upregulation of Hsp70 not only reduced extracellular oligomers but also reduced toxicity in in vitro studies [124]. Similarly, inhibition of Hsp90 also prevents release of extracellular α-synuclein via its interaction with endosome-associated protein Rab11a [127]. A prion-like propagation has also been reported recently [128]. Several proteins such as PrPC, LAG3, neurexin1, TLR2 and mGluR5 that function as receptors for the extracellular α-synuclein have been identified [129,130]. The pathological effects caused due to binding of α-synuclein to cell surface receptors is under investigation and is proposed to occur by various mechanisms (for a recent review, see [131]). Ca^2+^ dysregulation, synaptic dysfunction, altered glutamatergic synaptic transmission and α3-Na^+^/K^+^-ATPase activity as a result of extracellular α-synuclein have been reported [132,133]. Release of α-synuclein into the extracellular space as an alternative clearance mechanism when the cellular protein degradation mechanisms are impaired is also proposed [20]. Hence, an interplay between the intracellular degradation mechanism and cellular spreading can be envisaged. Extracellular chaperones such as clusterin (CLU) and α2-macroglobulin (α2M) bind to the hydrophobic region of α-synuclein and prevent the formation of larger aggregates. This interaction was reported to inhibit α-synuclein-induced membrane permeability and ROS production [134]. Strategies targeting extracellular release and spread of the toxic protein to adjacent neurons are envisaged as one of the efficient treatment options to be pursued.

## 3. Other PD-Related Proteins and Chaperones Regulating Them

Several genes other than *SNCA* have been identified that contribute to the development of PD. *PRKN, PARK7, LRRK2, PINK1, POLG, ATP13A2, FBX07, GBA, PLA2G6, VPS35, DNAJC6, SYNJ1* and *VPS13C* have been reported with high confidence to be associated with PD [135]. In this section, chaperones and associated proteins involved in the proteostasis of the above PD-associated proteins will be discussed.

### 3.1. Parkin

**Figure 3:**
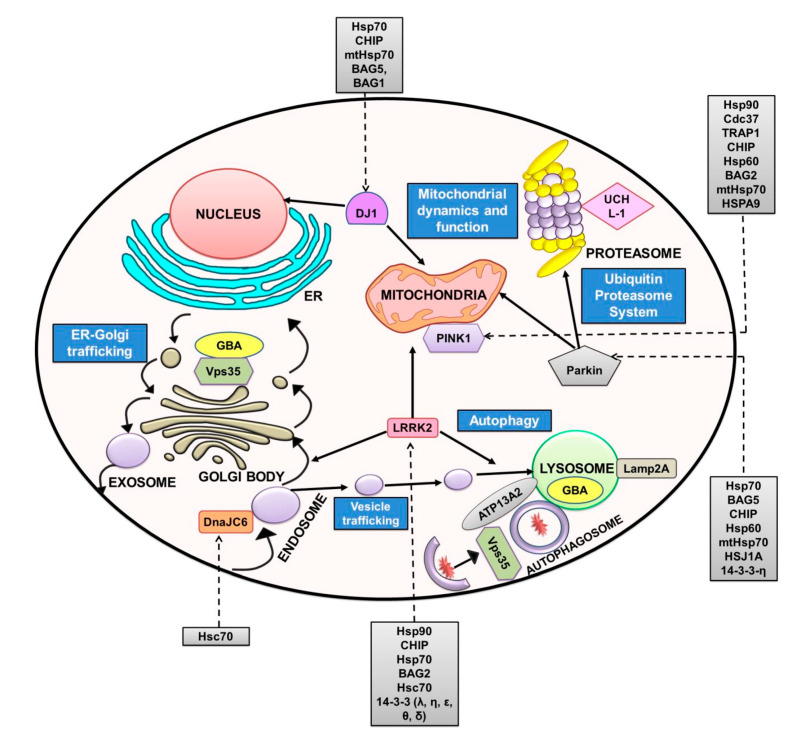
PD-related proteins and chaperones involved in proteostasis: Other well-characterized PD-related proteins, their cellular localization and functional pathways involved are depicted in this figure. These include leucine-rich repeat kinase 2 (LRRK2; PARK8), PTEN-induced putative kinase 1 (PINK1; PARK6), Parkin (PARK2), DJ-1 (PARK7), glucocerebrosidase (GBA), Vps35, UCH-L1 and ATP13A2. A role for LRRK2 in vesicular trafficking, mitochondrial dynamics and lysosome-autophagy pathways has been reported [206]. Mutations in LRRK2 associated with PD and perturb functions related to above cellular pathways are identified. PINK1/Parkin-mediated mitophagy has been extensively characterized, and mutations in these proteins are associated with PD. Parkin also induces protein degradation via UPS [207]. DJ-1 regulates mitochondrial function during oxidative stress and is also reported to localize to the nucleus [155,156]. Vps35 is a component of the retromer complex and has a role in endosomal trafficking and autophagy-mediated degradation [208]. GBA is an active lysosomal enzyme involved in the degradation of complex molecules and mutations in GBA are likely to accumulate toxic α-synuclein aggregates [208]. Another important protein is associated with lysosomal dysfunction, and therefore, cell viability is ATP13A2. Mutations in this protein are identified in PD patients [4]. Ubiquitin C-terminal Hydrolase L1 (UCH-L1) is involved in the UPS pathway of proteostasis, and mutations in this protein leading to PD pathogenesis have been identified [66]. Several DNAJ proteins that belong to the Hsp40 family are studied for their role in PD. Mutations in one of these chaperones, DNAJC6, resulted in the onset of juvenile PD [177]. Most of these proteins interact with chaperones to maintain their cellular homeostasis and.

*PRKN* encodes a 465-amino-acid, 52-kDa multifaceted E3 ubiquitin ligase called Parkin (Park2). It has a ubiquitin-like domain on its amino terminal followed by a 60-amino-acid linker and four zinc finger domains. Like other E3 ligases, the last three zinc finger domains of Parkin contain two RING domains flanking a cysteine-rich domain called in between RING fingers (IBR). The physiological function of Parkin in the regulation of mitophagy and the degradation of proteins has been widely accepted (Figure 3 [136,137]). Several substrates for the ubiquitination reaction of Parkin have been reported, with α-synuclein being one among them [138]. Mutations of Parkin associated with PD also exhibit a loss of its ubiquitin-ligase activity. Parkin was observed to localize to LBs in the substantia nigra region of the brain, highlighting the importance of its proteostasis in PD [139]. More than 100 mutations in *PRKN* have been associated with PD. The loss-of-function mutations in *PRKN* are reported to be the most common cause of autosomal recessive juvenile parkinsonism (AR-JP) and sporadic PD [140]. Interaction of Parkin with Hsp70, CHIP, a co-chaperone of Hsp70 and the ER-associated substrate of Parkin Pael-Receptor (Parkin-associated endothelin-like receptor) have been reported (Table 1). CHIP enhances Parkin-mediated ubiquitination of Pael-R by promoting dissociation of Hsp70 from the complex and positively regulates the E3 activity of Parkin in vivo [141]. In line with the above, Parkin was also reported to colocalize with Hsp70 [142]. The authors reported that Hsp70 promotes folding of the misfolded W453Stop mutant variant of Parkin [142]. Kalia and colleagues reported an interaction of BAG5 with Parkin and Hsp70 in HEK293T cells [143]. BAG5 inhibits the E3 activity of Parkin by enhancing its sequestration into protein aggregates similar to LBs. This was most likely due to the inhibition of Hsp70 function [143]. PD-associated mutations of Parkin such as K211N, T240R and G430D inhibit mitophagy, leading to the failure of selective mitochondrial removal, and most likely contribute to the pathogenesis of PD [144]. Yang and colleagues reported that mtHsp70/GRP75 interacts with Parkin and, together, regulates mitochondrial homeostasis. Knockdown of mtHsp70 resulted in abnormal accumulation of ROS and reduction in the copy number of mtDNA under induced stress conditions in HeLa cells [145]. Parkin was reported to relocate to the impaired mitochondria after membrane depolarization, thereby facilitating mitophagy. The RING domain mutant (C289G) of Parkin failed to induce mitophagy due to protein misfolding and resulted in the loss of localization to impaired mitochondria [146]. The cytosolic chaperone DNAJ family mitigated the aggregation of Parkin C289 variant in a Hsp70-dependent manner [147]. Expression of the neuronal co-chaperone HSJ1a (DNAJB2a) suppressed the misfolding of the mutant variant of Parkin C289G in SK-N-SH cells [146]. HSPJ1 also restored mitochondrial localization and function of Parkin mutants in mitophagy [146]. Interaction of Parkin with Hsp60 was also identified by proteomic analysis [148]. Interestingly, colocalization of Hsp60 with PINK1 and mtHsp70 was observed, suggesting a role for Hsp60 in PINK1/Parkin-mediated autophagy [149]. A role for the mitochondrial chaperone TRAP1 in the PINK1/Parkin pathway parallel to Parkin has been proposed. Enhanced expression of TRAP1 was reported to partially compensate for the loss of Parkin and vice versa in Drosophila [150]. Ubiquitin ligase activity of Parkin was negatively regulated by the chaperone-like protein 14-3-3η present abundantly in neurons. The role of this regulation in the pathogenesis of PD is not clearly understood [151].

### 3.2. PINK1

*PINK1* encodes a putative protein kinase comprising of 581 amino acids and a calculated molecular mass of 66 kDa. Upon proteolytic cleavage, a 55-kDa mature isoform is generated [144]. PINK1 is ubiquitously expressed in various tissues and has an N-terminal mitochondrial localization sequence (MTS) followed by a putative transmembrane region, a highly conserved serine/threonine kinase domain and a C-terminal regulatory sequence [152]. The physiological role of PINK1 in protection against stress-induced cell death and targeting of damaged mitochondria for degradation by PINK1/Parkin-mediated autophagy is well studied (Figure 3 [153]). PINK1 is the second most common factor associated with PD in which mutations are linked to the autosomal recessive inheritance of the disease [4]. Similar to Parkin, PINK1 was also found localized to LBs in PD brains [17] PINK1 interacts and forms a complex with the cytosolic chaperone Hsp90 and the co-chaperone Cdc37 [154]. The mutant variant of PINK1, L347P, could no longer interact with Hsp90 and Cdc37 and was reported to be less stable than the WT protein [155]. Interaction with the above chaperones stabilized the cleaved forms of the protein and facilitated their mitochondrial targeting [156]. Loss of interaction with Hsp90 and Cdc37 resulted in translocation of the full-length protein to mitochondria. A BAG family chaperone called BAG2 was reported to interact with PINK1 in HEK293T cells [157]. A role for BAG2 and CHIP in regulating the stability and levels of PINK1 has also been reported [157]. BAG2 was also reported to aid in the PINK1-dependent translocation of Parkin into mitochondria. Inhibition of the proteasomal pathway that prevents degradation of PINK1 or hindered mitochondrial import and subsequent processing of PINK1 have been proposed as the mode of action [157]. TRAP1 (Hsp75), a mitochondrial chaperone, is a substrate of PINK1 (kinase function) and is proposed to function downstream of PINK1 in Drosophila [150]. Expression of TRAP1 in PINK1 mutant Drosophila resulted in mitigation of PINK1 loss-of-function phenotype. TRAP1 also rescued mitochondrial defects in PINK1 mutant flies and attenuated the loss of dopaminergic neurons in mutant flies [150]. These results highlight the importance of chaperone-mediated therapies in the loss-of-function mutations associated with PD [158]. Further, interactions of PINK1 with the mitochondrial chaperones Hsp60, GRP75, LRPPRC and mtHsp70 has also been reported [149]. The levels of several of the interacting chaperones were reported to be reduced in PINK1-null cells [159].

### 3.3. DJ-1 

DJ-1 is a highly conserved 189-amino-acids cysteine protease ubiquitously present in all tissues [160]. The tertiary structure of DJ-1 adopts the α/β fold, and the monomer comprises of seven β-strands and nine α-helices [161]. In solution, it forms a dimer through interactions between helices and strands between each monomer. It is a multifunctional protein that has transcriptional regulation activity, antioxidant stress reaction and chaperone activity besides its protease function [162]. DJ-1 was originally identified as an oncogene, but later, loss of function mutations in the protein were found to be associated with familial PD of autosomal recessive inheritance [163]. Interestingly, DJ-1 was observed to be present in astrocytes in human brain and the association of oxidized DJ-1 with LBs in PD brains was reported [164,165]. Several mutant variants of DJ-1 are associated with PD, and some of these mutations disrupt the dimeric form of the protein [112]. DJ-1 also interacts with chaperones such as Hsp70, CHIP and mtHsp70. These chaperones are reported to interact with both WT and mutant variants such as L166P and M26I in human 293T cells [166]. Chaperone interaction was reported to be essential for the cytoplasmic and nuclear localization of DJ-1 (Figure 3) [166]. Localization of DJ-1 dimers to the mitochondria especially under oxidative stress has also been reported (Figure 3). However, mutant variants of DJ-1 localized to mitochondria were identified as monomeric units [167]. The mutant forms of DJ-1 (L166P and M26I) also exhibit strong interaction with chaperones when compared to the WT protein. Colocalization of DJ-1 with BAG5 in the cytoplasm of HEK293 cells was observed. BAG5 reduces the stability of DJ-1 by negatively influencing its dimerization and by reducing DJ-1 dimers in mitochondria that negatively affects mitochondrial protection [168]. Interestingly, this effect on DJ-1 caused by BAG5 was reported to be reversed by Hsp70 overexpression [168]. Another BAG chaperone, BAG1, also interacts with DJ-1 and restores the mutant variant (L166P) dimerization, activity and subcellular localization [169]. A co-immunoprecipitation study in SH-SY5Y cells showed that Parkin interacts with the mutant variants of DJ-1 and is present in a large protein complex that includes Hsp70 and CHIP. This interaction of Parkin with DJ-1 mutants such as L166P was proposed to aid in the monoubiquitination and not in polyubiquitination, thereby increasing the stability of the protein and not degradation. Such an interaction further serves for the pathogenesis of PD [170].

### 3.4. LRRK2 

*PARK8* encodes a 2527-amino-acid protein called LRRK2 or dardarin and is associated with sporadic and familial PD. It is a large multi-domain protein that belongs to the Roco superfamily of proteins and is expressed in many tissues [171]. It contains four protein–protein interaction domains called armadillo repeats (ARM), ankyrin repeats (ANK), leucine-rich repeats (LRR), Ras of complex (ROC) or C-terminal of ROC (COR). The kinase domain (KD) and the Trp-Asp-40 (WD40) domain confer enzymatic activities to the protein [25]. An interesting feature of the protein is the presence of both GTPase and kinase enzymatic activities [172]. It is reported to be localized to various intracellular structures such as endosomes, mitochondria, lysosome, plasma membrane, ER, Golgi and synaptic vesicles, and henceforth, several mechanisms of its causal effect on the pathogenesis of PD are also identified (Figure 3 [173]). However, it is still not clearly understood how this protein affects the pathobiology of α-synuclein. Mutations in LRRK2 are reported as one of the most common causes of late onset PD which follows autosomal dominant inheritance [101]. These mutations are reported to increase the kinase activity of the protein, and hence, identification of inhibitors against this protein or mechanisms of reducing its activity are considered good therapeutic options [174]. Autophosphorylation of LRRK2 at Ser-1292 and Rab protein phosphorylation were reported to be enhanced in the mutated variants [175]. G2019S mutation is the most common one and represents almost 4% of familial PD cases [176]. Chaperones like Hsp90, Cdc37 and Hsp70 were reported to interact with LRRK2, and a role for Hsp90 in maintaining the stability of LRRK2 is well documented [177]. Hsp90 stabilizes LRRK2, and upon inhibition of Hsp90, proteasomal degradation of LRRK2 was reported [177]. CHIP ubiquitinates LRRK2, and an interaction of the ROC domain of LRRK2 with TPR domain of CHIP in SH-SY5Y cells was reported [178]. In accordance with above findings, Ding and colleagues also reported that overexpression of Hsp90 stabilizes LRRK2 and attenuates the CHIP-mediated degradation of LRRK2 [179]. Interaction of BAG2 and Hsc70 with LRRK2 has been reported in human cells, and the homologues of these proteins are also reported to interact with the homologue of LRRK2 in *Caenorhabditis elegans* [180]. Inhibition of the CMA pathway due to the mutant variants of LRRK2 may further lead to toxicity caused by the accumulation of α-synuclein [181,182]. LRRK2 was also reported to interact with 14-3-3 via its phosphorylated Ser-910 and Ser-935 residues. PD-associated mutant variants of LRRK2 suppress Ser-910 and Ser-935 phosphorylation, thereby reducing the binding of 14-3-3. Loss of interaction between LRRK2 and 14-3-3 resulted in an increased accumulation of the protein in inclusion bodies [183]. However, the exact mechanism of how this protein influences LRRK2 stability and its relevance to PD is unknown [183]. 

### 3.5. DNAJ

The bacterial DNAJ family and the homologous Hsp40 proteins function as co-chaperones for Hsp70. This family of proteins consists of one to four domains and share a consensus 70-amino-acid J domain [184]. These proteins transiently associate with unfolded proteins that are delivered to Hsp70. The mechanism of action of these proteins is mentioned in Section 2.2 [185]. Three subtypes of the family depending on their domain composition (classes I, II or III, also called A, B or C) have been proposed [186]. Several class C proteins such as DNAJC5/CSPα, DNAJC13/RME-8, DNAJC12/JDP-1, DNAJC6/Auxilin-1 and DNAJC10/ERdj5 are implicated in PD [185]. The neuron-specific isoform of DNAJC6 called Auxilin-1 regulates clathrin-mediated endocytosis. It is a 100-kDa protein and is linked to AR-JP [187]. Auxilin-1 consists of three domains: the N-terminal phosphoinositide phosphatase (PTEN)-like domain, the central clathrin-binding domain and a C-terminal J domain [185]. The interaction of Auxilin-1 with Hsc70 through its J domain has been reported (Figure 3) [188]. This interaction stimulates the ATPase activity of Hsc70, which enhances the process of uncoating clathrin-coated vesicles (CCVs) [188]. Auxilin-1 first binds to CCVs through its clathrin-binding domain, and Hsc70 is subsequently recruited to the CCVs in presynaptic vesicles. Thus, it was established that Auxilin-1 and Hsc70 mediate uncoating of CCVs in neurons [189]. Several loss of function mutations have been identified in Auxilin that are associated with juvenile or early-onset PD [190]. An interaction between auxilin and LRRK2 has recently been reported in dopaminergic neurons generated using patient-derived induced Pluripotent Stem Cells (iPSCs) [191]. This interaction mediates phosphorylation of auxilin necessary for binding to clathrin. On the other hand, overexpression of auxilin also restored the phenotype of LRRK2 PD patient neurons to a certain extent [191]. 

A role in clathrin dynamics and, therefore, in the pathogenesis of PD has been proposed for multiple DNAJC proteins listed above. However, further investigations are necessary to shed light on the mechanism of action of these proteins in this pathogenesis of PD [190].

## 4. Conclusions

Research till now confirms that PD is not a single factor disease but has multiple factors associated to it and, at the same time, that the interactions between environmental factors and the genetic makeup of the person is very crucial. It is an age-associated neurodegenerative disorder and is majorly characterized by loss of proteostasis that eventually results in accumulation of toxic protein aggregates. Several proteins linked to the disease and the chaperones and associated co-chaperones involved in the proteostasis have been discussed in this manuscript. α-synuclein is the most important protein for which misfolding and aggregation into LBs is a characteristic feature of the disease. These protein aggregates hamper several essential cellular processes and subsequently affect neuronal viability. Treatment strategies targeting chaperones involved in PD that can restore the homeostasis of the target protein is also gravely explored. Development of patient-specific iPSC models of PD with the aid of novel genome editing tools has been instrumental in understanding the disease. However, to date, there is no cure to this disease and treatment given is only at alleviating the symptoms. As cellular structures are dynamic entities, a holistic approach at various factors in the pathogenesis is needed. An approach to look at both lipid and protein homeostasis together is essential to understand any interplay. Intracellular stress caused to organelles such as mitochondria and ER due to various aging-associated factors is also an important contributor to the disease. In this context, organelle interactions and association of the various proteins to different cellular structures may also shed more light on the molecular details. Posttranslational modifications of the PD-associated proteins and effect of these on protein–chaperone interactions and homeostasis of the proteins are also avenues that can shed light on the pathogenesis of the disease. The full functional relevance of several of these modifications and their interactions in both the physiological and pathological contexts is yet to be deciphered in detail.

## Figures and Tables

**Table 1:** Chaperones identified to date that regulate proteostasis of PD-related proteins: The table gives details regarding the cellular localization of the chaperones/co-chaperones and their function in proteostasis of PD related proteins. ER—Endoplasmic Reticulum, UPS—Ubiquitin Proteasome system, and CMA—Chaperone-Mediated Autophagy.

	PD Associated Protein	Associated Chaperones and Co-Chaperones	Function in Proteostasis	Localization	Reference
1.	α-synuclein (PARK1)	Hsp70 (HspA)	Refolding of misfolded α-synuclein and aids in degradation	Mitochondria, Cytosol, ER, Nucleus	[39,61]
		Hsp40 (DNAJ)	Refolding of misfolded α-synuclein	Cytosol, Nucleus	[61]
		DNAJB6	Prevents formation of α-synuclein fibrils and is involved in its degradation via UPS	Cytosol, Nucleus	[83]
		ST13 (Hip)	Co-chaperone of Hsp70	Cytosol	[57]
		BAG1 (Hap, Rap46)	Co-chaperone of Hsp70 and Hsc70; prevents aggregation	Cytosol, Nucleus	[58]
		BAG5	Co-chaperone of Hsp70 and Hsc70; role in of α-synuclein degradation	Cytosol, Nucleus, Mitochondria	[76]
		HSPA8 (Hsc70)	Prevents formation of α-synuclein fibrils and is involved in its degradation via CMA	Cytosol, Nucleus, Lysosomes, Plasma membrane	[62]
		HSPA9 (mtHsp70/GRP78/Mortalin)	Prevents formation of α-synuclein fibrils	Mitochondria	[74]
		BAG3	Co-chaperone of Hsp70; involved in the degradation of α-synuclein via macroautophagy	Cytosol, Nucleus, Plasma membrane	[118]
		Hsp90 (HspC)	Modulates assembly of α-synuclein to form fibrils	Cytosol, ER, Nucleus, Lysosome	[29]
		Aha1 (p38)	Co-chaperone of Hsp90; involved in α-synuclein fibrillation	Cytosol, ER,	[21]
		TRAP1 (HspC5)	Prevents formation of α-synuclein fibrils	Mitochondria	[33]
		p23	Co-chaperone of Hsp90; prevents formation of α-synuclein fibrils	Cytosol, Nucleus	[21]
		STI1 (Hop)	Co-chaperone of Hsp90; prevents formation of α-synuclein fibrils	Cytosol, Nucleus	[21]
		CHIP (Stub1)	Co-chaperone of Hsp90 and Hsp70; involved in degradation of α-synuclein	Cytosol, Nucleus	[21,117]
		Sgt1	Co-chaperone of Hsp90; prevents aggregation of α-synuclein	Cytosol, Nucleus	[21]
		CHP-1	Co-chaperone of Hsp90; involved in aggregation of α-synuclein	Cytosol	[21]
		Hsp60 (HspD1)	Prevents α-synuclein aggregation	Mitochondria, Cytosol	[64]
		DJ-1 (PARK7)	Inhibits α-synuclein glycation and aggregation	Mitochondria, Nucleus Cytosol	[67]
		Hsp27 (HspB)	Helps in disaggregation of α-synuclein fibrils and prevents aggregation	Cytosol, Nucleus, Cytoskeleton	[37]
		αB-crystallin (HSPB)	Helps in disaggregation of α-synuclein fibrils prevents aggregation	Nucleus, Cytosol	[36]
		HspB8	Helps in disaggregation of α-synuclein fibrils prevents aggregation	Cytosol, Nucleus	[38]
		HspB2B3	Helps in disaggregation of α-synuclein fibrils prevents aggregation	Cytosol, Nucleus	[38]
		Apg2 (Hsp110)	Helps in disaggregation of α-synuclein fibrils prevents aggregation	Nucleus, Cytosol	[40]
		14-3-3-θ	Involved in protein trafficking and refolding of α-synuclein	Nucleus, Cytosol	[66]
		proSAAS/7B2	Prevents formation of α-synuclein fibrils		[43]
		Torsin-1A (Tor1A)	Prevents formation of α-synuclein fibrils	Cytoskeleton, Nucleus, ER	[42]
		HDJ1	Prevents formation of α-synuclein fibrils	Cytosol, Nucleus	[42]
		HDJ2	Prevents formation of α-synuclein fibrils	Cytosol, Nucleus	[42]
		Atox1	Prevents formation of α-synuclein fibrils	Cytosol	[50]
		Sigma-1	Prevents formation of α-synuclein fibrils	Nucleus, ER	[47]
		Munc 18-1	Prevents formation of α-synuclein fibrils	Plasma membrane, Nucleus, Cytosol	[45]
		Arylsulfatase A (ARSA)	Prevents formation of α-synuclein fibrils	Lysosome, Cytosol	[53]
		Clusterin (CLU)	Prevents formation of α-synuclein fibrils	Extracellular, Mitochondria, Nucleus	[134]
		α2-macroglobulin (α2M)	Prevents formation of α-synuclein fibrils	Extracellular, Cytosol	[134]
2.	Parkin ( PARK2)	Hsp70 (HspA)	Refolding of misfolded Parkin	Mitochondria, Cytosol, ER, Nucleus	[142,169]
		BAG5	Co-chaperone of Hsp70 and Hsc70; inhibits E3 ubiquitin activity of Parkin	Cytosol, Mitochondria, Nucleus	[143]
		CHIP	Co-chaperone of Hsp70; enhances the E3 activity of Parkin through dissociation of Hsp70 from Parkin-Pael-R complexes	Cytosol, Nucleus	[141,169]
		mtHsp70 (Mortalin/HspA9)	Helps in maintaining mitochondrial homeostasis through its interaction with Parkin	Mitochondria	[146]
		HSJ1a	Co-chaperone of Hsp70; reduces misfolding and aggregation of mutant Parkin	Nucleus, Cytosol, ER	[146]
		14-3-3-η	Negatively regulated E3 ubiquitin ligase activity of Parkin	Mitochondria	[151]
		Hsp60 (HspD1)	Involved in mitochondrial protein folding	Mitochondria, Cytosol	[148]
3.	DJ-1 (PARK7)	Hsp70 (HspA)	Stabilizes DJ-1 and facilitates its protease activity	Mitochondria, Cytosol, ER, Nucleus	[166]
		CHIP	Facilitates protease activity of DJ-1	Cytosol, Nucleus	[166]
		mtHsp70	Facilitates protease activity of DJ-1	Mitochondria	[166]
		BAG1	Co-chaperone of Hsp70; stabilizes DJ-1 and facilitates activity	Cytosol, Nucleus	[169]
		BAG5	Reduces DJ-1 dimerization and attenuates its stability	Cytosol, Mitochondria, Nucleus	[168]
4.	PINK1 (PARK6)	Hsp90 (HspC)	Stabilization of cleaved forms of PINK1	Cytosol, ER, Nucleus, Lysosome	[156]
		Cdc37	Co-chaperone of Hsp90; stabilizes Pink1	Cytosol	[156]
		TRAP1 (HSPC5)	Restores Pink1 loss-of-function phenotypes	Mitochondrion, Nucleus	[150]
		CHIP	Decreases ubiquitinated Pink1	Cytosol, Nucleus	[157]
		Hsp60 (HspD1)	Involved in mitochondrial protein folding	Mitochondria, Cytosol	[149]
		BAG2	Regulates the level of Pink1	Cytosol, Nucleus	[157]
		mtHsp70 (HspA9 GRP75/Mortalin)	Regulates Pink1-Parkin dependent homeostasis of mitochondrial proteins	Mitochondria, Nucleus	[149]
		LRPPRC	Regulates Pink1-Parkin dependent homeostasis of mitochondrial proteins	ER, Nucleus, Mitochondria, Cytoskeleton	[149]
5.	LRRK2 (PARK8)	Hsp90 (HspC)	Stabilization of LRRK2	Cytosol, ER, Nucleus, Lysosome	[177]
		Cdc37	Co-chaperone of Hsp90; stabilizes LRRK2	Cytosol	[177]
		CHIP	CHIP mediated proteasomal degradation of LRRK2	Cytosol, Nucleus	[178]
		Hsp70	Proteasomal degradation of LRRK2	Mitochondria, Cytosol, ER, Nucleus	[177]
		Hsc70	Proteasomal degradation of LRRK2	Plasma membrane, Cytosol, Nucleus, and Lysosome	[177]
		14-3-3	Interacts with LRRK2 and regulates its kinase activity	Mitochondria, Cytosol, Plasma membrane, Nucleus	[183]
		BAG2	Interacts with LRRK2	Cytosol, Nucleus	[180]
6.	DnaJC6/Auxilin (PARK9)	Hsc70	Clathrin-mediated endocytosis	Plasma membrane, Cytosol, Nucleus, and Lysosome	[183]
